# Does contralateral knee range of motion predict postoperative knee range of motion after total knee arthroplasty?

**DOI:** 10.1186/s43019-020-00044-5

**Published:** 2020-06-05

**Authors:** Robert R. Burnham, Samantha E. Bialek, Amy Wozniak, Nicholas M. Brown

**Affiliations:** 1 Department of Orthopaedic Surgery & Rehabilitation, Loyola University Medical Center, 2160 S. First Avenue, Maguire Suite 1700, Maywood, IL 60153 USA; 2 Stritch School of Medicine, Loyola University Chicago, 2160 S. First Avenue, Maywood, IL 60153 USA; 3 Center for Translational Research, Loyola University Chicago, 2160 S. First Avenue, Maywood, IL 60153 USA

**Keywords:** Total knee arthroplasty, Contralateral range of motion, Postoperative range of motion

## Abstract

**Purpose:**

The aim of this study was to determine if contralateral knee range of motion is associated with postoperative range of motion in the operative knee after total knee arthroplasty.

**Methods:**

Contralateral (nonoperative) knee range of motion was compared to postoperative knee range of motion after total knee arthroplasty using linear regression models in 59 patients who had undergone primary total knee arthroplasty with a minimum of 4 months postoperative follow-up data (range 4–13 months).

**Results:**

A strong linear relationship was observed between contralateral knee ranges of motion of 115° or greater and postoperative knee ranges of motion after total knee arthroplasty (slope 0.93, 95% CI 0.58–1.29, *P* < 0.0001), with a mean difference of −7.44° (95% CI −10.3 to −4.63, *P* < 0.0001). However, there was no association between contralateral knee range of motion and postoperative knee range of motion when contralateral knee range of motion was less than 115°.

**Conclusion:**

Contralateral knee range of motion of 115° or greater correlates linearly with postoperative range of motion after total knee arthroplasty, and thus may be predictive in such cases.

## Introduction

An important goal of total knee arthroplasty (TKA) is to achieve satisfactory postoperative range of motion (ROM) for the patient, as ROM is an important outcome measure of TKA [1–4] and is the main component of most knee scoring systems [5, 6]. Several studies have sought to determine predictive factors of postoperative ROM after TKA, and this is an area of active research [4, 7–9].

Of note, Ritter et al. [1] retrospectively studied 3066 patients (4727 knees) with primary TKA using statistical clustering, log-linear regression and regression tree analysis, and found the strongest predictor of postoperative ROM after TKA by far to be preoperative ROM, regardless of preoperative alignment. While some statistically significant relationships were found with reduced postoperative flexion and other factors such as female sex, intraoperative flexion and preoperative tibiofemoral alignment, they concluded these statistical relationships bore little to no clinical significance. Anouchi et al. [3] also included these factors in addition to previous surgery and modification of posterior femoral condyle geometry in their analyses through a multicenter prospective study with 621 patients and found only preoperative ROM and scores to be predictive of post-TKA ROM. The predictive value of posterior cruciate ligament status for post-TKA ROM has also been debated in the literature but has also not been shown to be a predictive factor [10]. Similarly, implant design and insert type have not been shown to affect postoperative ROM [11–16]. Several other studies have evaluated a variety of demographic and comorbidity-related factors that have shown to have some but variable predictive value [4, 8, 9, 17, 18].

To date, the factor that has consistently been shown in the literature and is well established to predict postoperative ROM is preoperative ROM in the same knee [4, 8, 9, 19–21]. Preoperative flexion has consistently been shown to be the strongest predictor of postoperative flexion [1, 2, 4, 10, 12, 22]. Other studies on post-TKA outcomes have expanded to include the contralateral, nonoperative knee in analyses. Such studies have evaluated contralateral knee osteoarthritis, pain and biomechanics to predict the need for contralateral TKA after primary TKA [23, 24]. At our institution, we have observed post-TKA outcomes in the operative knee such as ROM, flexion contracture and need for manipulation under anesthesia (MUA) which appear to eventually match that of the preoperative contralateral, nonoperative knee in our patients. Patients with better baseline contralateral knee ROM tend to achieve similar post-TKA ROM in the operative knee, and those with less baseline ROM in the contralateral knee tend to have worse post-TKA ROM. These observations have thus led us to hypothesize that contralateral knee ROM may be predictive for postoperative knee ROM after TKA.

While studies on post-TKA outcomes and predictors have begun to include various evaluations of the contralateral knee [23–26], to our knowledge there are no studies in the literature to date on whether ROM in the contralateral, nonoperative knee is predictive of postoperative ROM in the operative knee after TKA. Thus, the purpose of the present study was to determine if postoperative knee ROM after TKA can be predicted by a patient’s preoperative ROM in the nonoperative knee by comparing postoperative ROM arc measurements after TKA to preoperative ROM arc measurements in the nonoperative knee.

## Methods

### Patients

Following Institutional Review Board approval, all patients who had undergone a primary TKA performed by one surgeon at our institution between September 2017 and June 2019 were initially considered for inclusion in this retrospective study (*n* = 84). Because 12 of those patients underwent staged bilateral TKAs performed within 2 to 13 months of each other, only postoperative data from the second surgery was included in statistical analyses (*n* = 72) and the postoperative data from the first surgery was excluded (*n* = 12). Of the 72 patients, only those with a minimum of 4 months postoperative follow-up data (range from 4–13 months) were included in statistical analyses for ROM (*n* = 59). ROM measurements of bilateral knees, including flexion, extension and ROM arc, were recorded pre- and postoperatively. Analyses for outcomes of flexion and manipulation had varying sample sizes depending on missing data. The presence or absence of a flexion contracture, the need for postoperative MUA, implant type, age, sex and type of preoperative osteoarthritis (varus, valgus, neutral) were also recorded and included in statistical analyses. Preoperative ROM in the contralateral, nonoperative knee was compared to postoperative ROM arc in the operative knee.

### Statistical methods

**Fig. 1:**
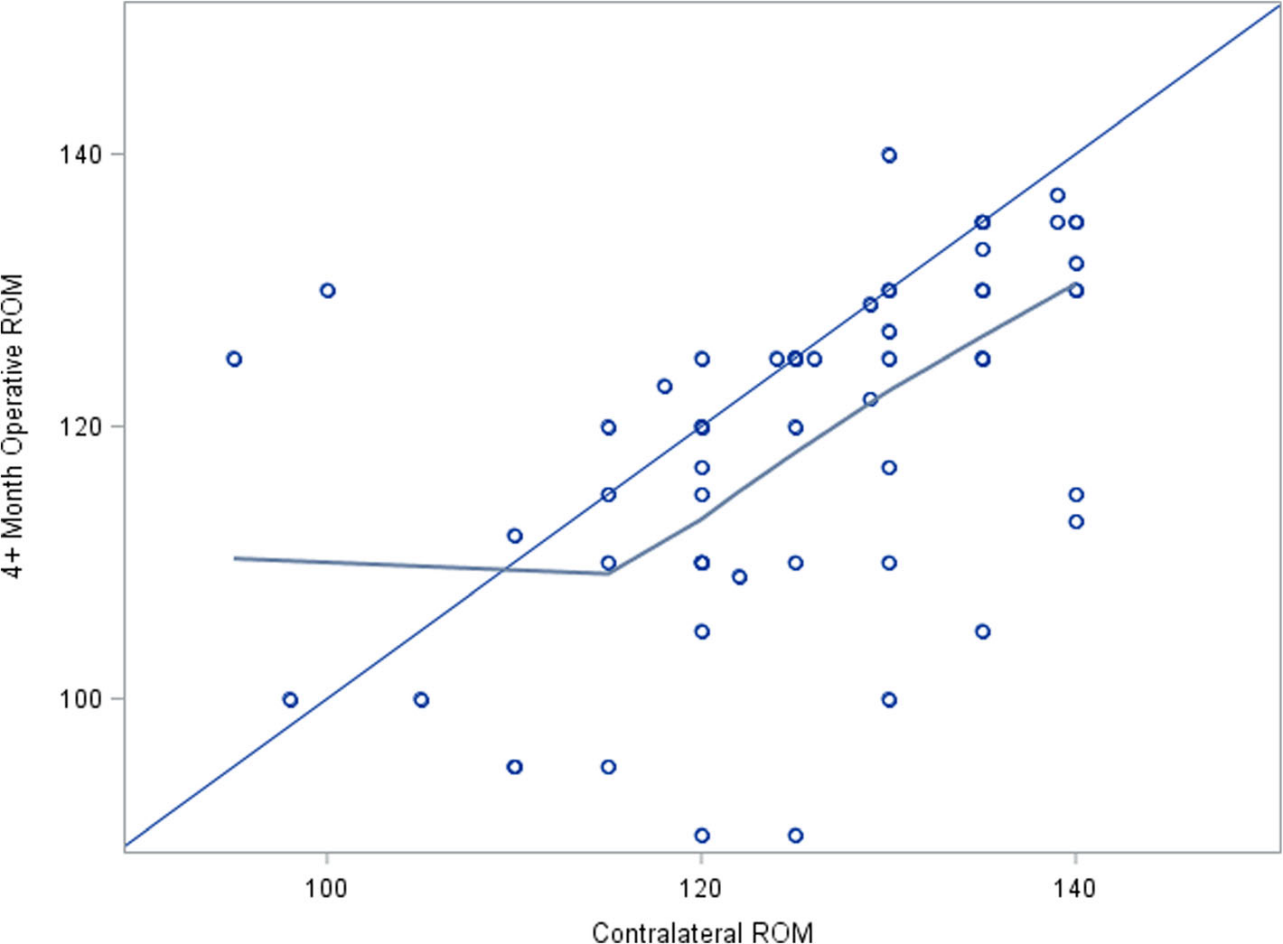
Regression model with spline. Relationship between post-total knee arthroplasty (TKA) range of motion (ROM) at final follow-up and contralateral ROM of <115° and ≥115°. Dark blue line: LOESS curve illustration of relationship between contralateral ROM and post-TKA ROM at final follow-up. Light blue line: reference line where contralateral ROM is perfectly predictive of post-TKA ROM at final follow-up

Scatter plots and LOESS curves were used to evaluate the linear relationship between contralateral knee ROM and postoperative ROM in the operative knee at final follow-up. The results illustrated a piecewise linear relationship with a knot at 115°. Thus, regression models with a spline were used to evaluate the association of contralateral knee ROM and postoperative ROM for the operative knee at final follow-up below contralateral ROM of 115° and above contralateral ROM of 115° (Fig. 1). Differences between a priori risk factors were assessed using interaction terms of slope prior to ROM of 115° and greater than 115°. Fisher exact tests were used to test the association of contralateral knee flexion contracture with postoperative knee flexion contracture at final follow-up and MUA, and Fisher exact tests were used to test the association for contralateral ROM and MUA.

## Results

The average age of the patient population studied was 62.8 years (standard deviation = 9.4), 58% of which were female and 42% male. Overall, 75% of patients had preoperative varus osteoarthritis and 82% of patients had a posterior-stabilized (PS) implant (Table 1).

**Table 1:** Patient characteristics, *n* = 72

Patient characteristic	***n*** (%) or mean (SD)
Male	30 (42)
Female	42 (58)
Age (years)	62.8 (9.4)
Varus	54 (75)
Valgus	7 (10)
Neutral	11 (15)
CR	13 (18)
PS	59 (82)

*CR* cruciate retaining, *PS* posterior stabilized, *SD* standard deviation

At a contralateral ROM of 115° or greater there was a strong linear relationship observed between contralateral ROM and postoperative ROM (slope 0.93, 95% confidence interval (CI) 0.58–1.29; *P* < 0.0001; Table 2). For every unit increase in a contralateral knee ROM of 115° or greater, there was a 0.93° increase in postoperative ROM in the operative knee. On average, for values greater than 115°, the difference in postoperative knee ROM tended to be −7.44 (95% CI −10.3 to −4.63; *P* < 0.0001) compared to contralateral knee ROM (Table 3) (Fig. 1). However, there was no association when the contralateral ROM was less than 115°. Results were similar for all subgroups and there were no differences between subgroups for any of the other predictors studied (Tables 2 and 3).

**Table 2:** Outcome: postoperative ROM at final follow-up for operative knee; predictor: contralateral ROM, *n* = 59

Predictor	Association prior to 115°, slope (95% CI)	***P*** value	Association from 115°, slope (95% CI)	***P*** value	Difference between groups, ***P*** value
Contralateral ROM, all patients	−0.32 (−1.1, 0.43)	0.4016	0.93 (0.58, 1.29)	<0.001	NA
Contralateral ROM, male	0.47 (−0.88, 1.83)	0.4841	0.99 (0.48, 1.51)	0.0003	0.7664
Contralateral ROM, female	−0.61 (−1.55, 0.32)	0.1951	0.89 (0.37, 1.40)	0.0010	
Contralateral ROM, age <63	−0.84 (−2.16, 0.49)	0.2088	1.14 (0.66, 1.61)	<0.0001	0.2158
Contralateral ROM, age ≥63	0.03 (−0.91, 0.97)	0.9481	0.68 (0.10, 1.25)	0.0216	
Contralateral ROM, varus	−0.90 (−1.78, − 0.02)	0.0445	0.84 (0.44, 1.24)	0.001	0.9119
Contralateral ROM, valgus	NE		0.92 (−0.41, 2.25)	0.1723	
Contralateral ROM, neutral	NE		NE		
Contralateral ROM, CR	NE		1.344 (0.49, 2.19)	0.0025	0.2924
Contralateral ROM, PS	−0.23 (−1.01, 0.55)	0.5569	0.85 (0.44, 1.25)	<0.0001	

*CI* confidence interval, *CR* cruciate retaining, *NA*, not applicable, *NE* not estimated (due to small sample size), *PS* posterior stabilized, *ROM* range of motion

**Table 3:** Difference in contralateral and postoperative ROM where contralateral ROM ≥115, *n* = 52

Predictor	***n***	Mean (SD) ROM of Operative Knee	Mean (SD) ROM of Contralateral Knee	Difference (95% CI)	Difference between groups, ***P*** value
Contralateral ROM, all patients	52	120.8 (12.1)	128.3 (8.0)	−7.44 (−10.3, −4.63)*	NA
Contralateral ROM, male	22	121.6 (13.0)	128.6 (9.1)	−7.00 (−11.2, −2.8)*	0.7857
Contralateral ROM, female	30	120.3 (11.5)	128.0 (7.2)	−7.77 (−11.8, −3.7)*	
Contralateral ROM, age <63^a^	26	120.8 (12.6)	129.0 (8.9)	−8.2 (−11.65, −4.7)*	0.5981
Contralateral ROM, age ≥63^a^	26	120.9 (11.7)	127.6 (7.1)	−6.7 (−11.3, −2.0)*	
Contralateral ROM, varus	39	118.9 (12.1)	127.1 (8.1)	−8.3 (−11.4, −5.1)*	0.3408
Contralateral ROM, valgus	6	127.8 (8.1)	127.5 (6.9)	0.3 (−12.6, 12.23)	
Contralateral ROM, neutral	7	125.9 (12.6)	135.4 (4.5)	−9.6 (−20.9, 1.77)	
Contralateral ROM, CR	10	120.6 (14.1)	129.1 (8.4)	−8.5 (−14.9, −2.1)*	0.6412
Contralateral ROM, PS	42	120.9 (11.7)	128.1 (8.0)	−7.2 (−10.4, −3.9)*	

*CI* confidence interval, *CR* cruciate retaining, *NA*, not applicable, *PS* posterior stabilized, *ROM* range of motion, *SD* standard deviation

^a^Dichotomized at median age

*Significantly different from 0 (*P* < 0.05)

Six of nine (66%) patients with a contralateral knee flexion contracture lacked full extension on their operative knee compared to 13 of 49 (26%) without a contralateral flexion contracture (*P* = 0.0496).

There were only five patients in this cohort who, due to significant postoperative stiffness, were indicated for postoperative MUA after their TKA, which made comparisons difficult. Among these five patients, MUA was performed at a mean of 9 weeks post-TKA. However, contralateral ROM was not associated with the need to perform postoperative MUA (0/9 (0%) ROM <115° versus 5/63 (7.94%) ROM ≥115°; *P* = 1.000), nor was contralateral flexion contracture (1/9 (11%) flexion contracture versus 4/61 (7%) no flexion contracture; *P* = 0.509).

## Discussion

These results suggest that when the nonoperative, contralateral knee ROM is 115° or greater it may be used as a predictor for postoperative ROM after TKA. Additionally, the presence of a contralateral knee flexion contracture may predict postoperative flexion contracture in the operative knee after TKA. Patients mobilize in a relatively symmetric manner; thus, the contralateral knee influences the motion of the operative knee after TKA. As patients begin to walk, climb stairs, squat and sit with increased frequency postoperatively, as most do by final follow-up post-TKA, the contralateral knee continues to influence the ultimate motion of the operative knee. Excellent motion in one knee promotes excellent motion in the other knee, consistent with the threshold value of 115° suggested by our results.

At contralateral knee ROM less than 115° ROM is less optimal and its influence on ROM of the operative knee is more variable. While contralateral knee ROM less than 115° did not linearly correlate with post-TKA ROM, it is important to note contralateral ROM less than 115° did not limit the operative knee in achieving a functional ROM; all patients in this cohort achieved a functional ROM after TKA. While interpretation of these results and the proposed threshold value of contralateral knee ROM must take our sample size into consideration, the present study does offer the first attempt at predicting postoperative ROM after TKA according to preoperative contralateral knee ROM; a strong association at or above contralateral knee ROM of 115° was observed in this patient cohort.

As mentioned, preoperative ROM in the operative knee is well established as the best predictor of postoperative ROM after TKA [1, 2, 4, 8–10, 12, 19–22, 27]. In two recently developed clinical predictive models by Pua et al. [9] and Stratford et al. [7], preoperative ROM and scores are primarily used to predict postoperative knee measures, with little if any weight in their models placed on other statistically significant factors such as patient sex, age, body mass index, underlying disease, preoperative walking limitations and pain.

The literature on preoperative risk factors and predictors for post-TKA flexion contracture and need for MUA mirrors that of predictors for post-TKA ROM, with the strongest and most reliable predictor being the presence of a preoperative flexion contracture in the operative knee, not readily varied by other factors [1, 2, 9, 16, 19, 21, 28, 29]. Harato et al. [25, 26] have conducted gait and weight bearing studies in patients with flexion contractures and have delineated the abnormal forces placed on the contralateral knee in TK A[26] and non-TKA settings [25]. They have demonstrated that flexion contractures >15° after TKA promotes progression of osteoarthritis and the need for TKA in the contralateral knee but have not studied such an association with contralateral knee flexion contracture [30], as have no other studies in the literature to date. Our results suggest a possible association between the presence of any degree of contralateral knee flexion contracture and flexion contracture after TKA in the operative knee. Contralateral flexion contracture may thus serve as a predictive factor for post-TKA flexion contracture.

This study included PS versus cruciate retaining (CR) implants as a subgroup in our statistical analyses and we found similar results for all subgroups and no differences between subgroups, including PS versus CR (*P* = 0.6412). These results support those of the several studies (randomized controls [11], retrospective [15] and prospective [16] analyses and meta-analyses [12–14]) that have previously demonstrated implant type to have no significant effect on postoperative ROM after TKA, both statistically [11, 13, 15, 16] and clinically [11–16]. As an example, the meta-analysis of Bercik et al. [12] comparing PS versus CR in TKA concluded that while there may be a statistically significant difference in postoperative ROM favoring PS implants, the clinical importance of this is unknown and therefore the decision to use PS versus CR implants should still be based on surgeon preference and comfort. Other studies have more definitively demonstrated no difference between PS and CR on post-TKA ROM and have concluded that implant type lacks predictive value for post-TKA ROM [11, 13–16].

Limitations of the present study include its sample size and relatively short follow-up time. This must be considered when interpreting our results. Additionally, while there is a relatively high rate of patients that lacked full extension in this cohort, for many of these patients this is likely due to a relatively short postoperative follow-up time (between 4 months to 1 year); the lack of full extension in these patients was around 3–5°. Finally, there was a relatively high rate of MUA. The surgeon in this study has a historical rate of MUA of around 3%. It is possible that this higher rate was due either to a closer emphasis on measuring ROM in these patients, or to a statistical abnormality given our smaller sample size.

While the study is limited by sample size, given that data were used only from TKAs performed by one surgeon, a major strength is its elimination of inter-surgeon variability. The authors view this as somewhat of a trade-off for the smaller sample size. Another strength is the study’s novelty—this hypothesis has not been tested before in the literature to date. The study offers a first attempt at using contralateral knee ROM to predict postoperative ROM after TKA that may be confirmed in future studies with larger sample sizes and longer postoperative follow-up.

Since the predictive value of many patient factors that have been studied to date for postoperative ROM after TKA remains low [3, 7, 8, 10–16] or variable [9, 17], preoperative ROM in the operative knee is most often used to predict postoperative ROM after TKA [4, 8–10, 22]. There remains a paucity of other reliable, clinically relevant and practical predictive factors. Thus, despite its limitations, the results of the present study may offer another strong clinical predictor to consider contralateral knee ROM when it is 115° or greater.

## Conclusion

Our results suggest that contralateral knee ROM has a strong positive association with postoperative ROM after TKA when contralateral knee ROM is 115° or greater. Additionally, our results suggest flexion contracture in the contralateral knee is predictive of postoperative flexion contracture in the operative knee after TKA. These results may thus serve as a predictor for postoperative ROM after TKA and may perhaps also be referenced in preoperative discussions with patients regarding anticipated post-TKA outcomes.

## Data Availability

The datasets generated and/or analyzed during the current study are available from the corresponding author on reasonable request.
